# Unveiling the shadows: Beyond the hype of AI in education

**DOI:** 10.1016/j.heliyon.2024.e30696

**Published:** 2024-05-03

**Authors:** Abdulrahman M. Al-Zahrani

**Affiliations:** Department of Learning Design and Technology, Faculty of Education, University of Jeddah, P.O. box 15758, 21454, Jeddah, Saudi Arabia

**Keywords:** AI-integration, Data privacy, Algorithmic bias, AI-transparency, AI-ethics, AI-reliability, AI-generated content

## Abstract

Despite the wave of enthusiasm for the role of Artificial Intelligence (AI) in reshaping education, critical voices urge a more tempered approach. This study investigates the less-discussed 'shadows' of AI implementation in educational settings, focusing on potential negatives that may accompany its integration. Through a multi-phased exploration consisting of content analysis and survey research, the study develops and validates a theoretical model that pinpoints several areas of concern. The initial phase, a systematic literature review, yielded 56 relevant studies from which the model was crafted. The subsequent survey with 260 participants from a Saudi Arabian university aimed to validate the model. Findings confirm concerns about human connection, data privacy and security, algorithmic bias, transparency, critical thinking, access equity, ethical issues, teacher development, reliability, and the consequences of AI-generated content. They also highlight correlations between various AI-associated concerns, suggesting intertwined consequences rather than isolated issues. For instance, enhancements in AI transparency could simultaneously support teacher professional development and foster better student outcomes. Furthermore, the study acknowledges the transformative potential of AI but cautions against its unexamined adoption in education. It advocates for comprehensive strategies to maintain human connections, ensure data privacy and security, mitigate biases, enhance system transparency, foster creativity, reduce access disparities, emphasize ethics, prepare teachers, ensure system reliability, and regulate AI-generated content. Such strategies underscore the need for holistic policymaking to leverage AI's benefits while safeguarding against its disadvantages.

## 1 Introduction

Artificial Intelligence (AI) has been the linchpin of revolutionary advancement in diverse sectors since its conceptualization in the mid-20th century. Its tendrils have extended into virtually every facet of modern life revolutionizing the way we communicate, manage data, conduct business, secure digital assets, and interact within social frameworks [1,2]. Now, as we navigate an era where our lives intertwine ever more closely with digital ecosystems, AI-based innovations have profoundly permeated the realm of education and research, altering our educational paradigms, methodologies, and institutional structures [[3], [4], [5], [6], [7]].

The academic lens has primarily focused on the optimistic spectrum of AI's intervention in the educational landscape, especially its capability to customize learning, its instrumental role in administrative automation, and the enhancement of pedagogic efficacy [1,[4], [5], [6], [7], [8], [9], [10], [11], [12], [13], [14]]. Yet, the prevailing discourse has tended to understate, or at times, elide the complexities and potential pitfalls that AI's integration brings to educational settings.

This study emerges from a critical need to address the dearth of research into the potentially adverse consequences of AI within educational contexts, a gap that constitutes a significant blind spot in our collective understanding. By adopting a counter-narrative approach, the research endeavors to pierce the prevailing facade of technological utopianism and expose the nuanced challenges AI introduces to the scholarly and educational ecosystems.

The exploration of AI's less celebrated nuances will traverse areas such as the disruption of traditional pedagogical relationships, potential biases encoded within educational algorithms, privacy concerns associated with student data management, and the exacerbation of digital divides. It aims to shine a light on AI's shadow side where unintended, and often overlooked, repercussions on cognitive development, social interactions, and learning autonomy reside. These are critical considerations that educational stakeholders must grapple with to harness AI's potential responsibly and ethically.

This inquiry is therefore grounded in a commitment to intellectual candor and rigour, seeking to balance the techno-optimism with sober reflection on the potential for technological overreach and its attendant risks. The ‘dark side’ explored herein is not to disavow AI's value but to espouse a mature vigilance over its pervasive influence. The study is designed as a conscientious exploration into AI's complex layers within the educational panorama, probing beyond surface level benefits to uncover deeper implications that could shape the future of education in profound and unpredictable ways.

The research delves into these uncharted waters propelled by a pivotal question that seeks to anchor the discourse within a critical framework.

• **RQ**: What are the potential negative implications of integrating AI in education beyond the optimistic narratives?

This study's scope thus selectively navigates the dichotomy of AI's promises versus its perils in the educational arena, while purposefully decoupling from an extensive analysis of AI's mechanics or enumerating its widely documented positive aspects. It is an exploratory endeavor to broaden the discourse, foster critical analysis, and advocate for a more reflexive adoption of AI in educational contexts, ensuring the technology serves as a tool for enhancement rather than an instrument for inequity.

## 2 2Background: critical perspectives on AI potential drawbacks in education

AI is often lauded for its advanced capabilities, substituting for human cognition in activities that entail complex thinking, problem-solving, and analytical reasoning [10]. AI systems strive to simulate nuanced human cognitive processes, encompassing critical thinking, comprehension, abstraction, and adaptive learning [10,13]. In the realm of education, AI's imprint can be found in a variety of technologies such as intelligent tutoring systems, adaptive learning environments, conversational agents, automated evaluative tools, and sophisticated data analytical instruments [[3], [4], [5], [6], [7], [8]]. These innovations herald a transformative era in education, offering tailored educational experiences, streamlining administrative functions, providing instantaneous feedback, and fostering more informed decision-making in educational strategies [[3], [4], [5], [6], [7], [8]].

Renowned organizations such as UNESCO (2021) highlight AI's potential in mitigating educational disparities and introducing cutting-edge methodologies in pedagogy. AI's capacity for personalization adapts learning pathways to individual student profiles, thereby boosting cognitive engagement and the consolidation of academic content [8,9,12]. Further, AI-facilitated data analysis lends educators potent tools to extract actionable insights from extensive student datasets, which in turn influences curriculum development and pedagogical techniques [8,14]. Additionally, the automation of routine administrative duties and the optimization of assessment procedures liberate educators from time-consuming tasks, thereby enriching their pedagogical engagements [8,12].

However, the influx of AI in educational settings is not without its pitfalls which necessitate critical scrutiny to foster both its responsible employment and equitable accessibility. The velocity of AI innovation outstrips the methodical progression of regulatory scrutiny and ethical debriefing, precipitating a spectrum of risks and ethical quandaries. Scholars like Guan et al. (2020) cast a spotlight on the burgeoning domain of student data analytics and profiling that impinges upon individual privacy, engenders discriminatory biases through algorithms, and could perpetuate social inequities. One proposed mitigative strategy is the formulation of interdisciplinary academic offerings that inculcate an awareness of AI's dual potential as both a tool for progress and a vector for challenges amongst future professionals [2].

A thorough dissection of the extant scholarly contributions reveals a series of underlying concerns associated with AI in educational contexts. These concerns crystallize around themes such as the diminishment of human-to-human engagement, the safeguarding of sensitive data, emerging security vulnerabilities, the opacity of algorithmic functions, the potential erosion of critical and creative faculties, the exacerbation of technological disparities commonly referred to as the digital divide, multifaceted ethical conundrums, the imperative for ongoing educator professional growth, dependency on the reliability and maintenance of AI systems, and the ethical implications surrounding AI-generated academic content. Such considerations commit to the forefront a nuanced labyrinth of challenges that must be navigated deftly to effectively integrate AI into educational organizations. See Table 1 for a detailed exposition.

**Table 1:** Content analysis of AI potential negative impacts and drawbacks.

Theme	Critical Perspective	References
1. Loss of Human Connection	The increased use of AI in education may result in a loss of human connection between students and educators, potentially impacting students' motivation, social development, and overall educational experience.	[[15], [16], [17], [18], [19], [20], [21]]
2. Data Privacy and Security Concerns	The use of AI often involves collecting and analyzing large amounts of student data. This raises concerns about data privacy, security breaches, and the potential misuse of sensitive information.	[[22], [23], [24], [25]]
3. Algorithmic Bias and Discrimination	AI algorithms can reflect societal biases present in their training data, leading to algorithmic bias and discrimination against marginalized student groups in education.	[[26], [27], [28], [29], [30], [31], [32], [33]]
4. Lack of Transparency and Explainability	The complexity and opacity of AI algorithms used in education can make it difficult to understand their decision-making process. This lack of transparency raises concerns about accountability and potential unfair treatment of students.	[[34], [35], [36], [37], [38], [39], [40]]
5. Reduced Critical Thinking and Creativity	Overreliance on AI in education may limit the development of students' critical thinking and creativity. If AI systems provide predefined answers and dictate the learning process, students may have fewer opportunities for independent problem-solving and creative exploration.	[[41], [42], [43]]
6. Unequal Access and Technological Divide	The integration of AI in education may widen the technological divide between students with access to advanced technologies and those without. Unequal access to AI resources could exacerbate educational inequalities between privileged and disadvantaged students.	[[44], [45], [46], [47], [48]]
7. Ethical Considerations in AI Use	The ethical implications of AI in education need to be carefully examined. Issues such as informed consent, data ownership, algorithmic accountability, and potential unintended consequences must be considered.	[[49], [50], [51], [52], [53], [54], [55]]
8. Teacher Professional Development and Role	The implementation of AI in education requires teachers to adapt their instructional practices and develop new skills. Inadequate training and support for teachers in using AI tools may hinder effective application.	[[56], [57], [58], [59], [60]]
9. Dependence on AI Reliability and Maintenance	Heavy reliance on AI systems for educational tasks raises concerns about their reliability and maintenance. Technical issues or system failures may disrupt learning processes and create dependence on AI systems that may not always be reliable or available.	[[61], [62], [63], [64], [65], [66]]
10. Implications of AI-generated Content	The use of AI to generate educational content, such as automated essay grading or content creation, raises questions about authenticity, intellectual property, and the value of human input in educational processes.	[41,[67], [68], [69], [70], [71]]

Comprehending the potential challenges and problems that accompany AI integration is crucial for stakeholders, policymakers, and educational practitioners. This study seeks to illuminate the negative aspects of AI to promote a balanced and informed integration strategy, while still harnessing its positive influences within the educational sphere. By confronting ethical issues and reducing risks, stakeholders will be positioned to ensure a responsible and advantageous application of AI technologies.

## 3 Methodology

The current research adopts a multi-phase sequential exploratory design, integrating conventional content analysis [72] with survey research to develop and validate a theoretical model that elucidates the negative implications of integrating AI in education. Conventional content analysis is typically employed within research designs aiming to clarify a phenomenon, particularly when there is limited existing theory or research literature on the subject. Instead of imposing predetermined categories, researchers opt to derive categories and their labels directly from the data [72].

### 3.1 Research design and data collection

The research design consisted of two sequential phases.

1. Development of a theoretical model through conventional content analysis:
  • Systematic review and analysis of the existing literature on the potential negative impacts of AI in education were conducted using conventional content analysis.
  • The literature search employed keywords like “AI in education,” “negative impacts of AI,” “drawbacks of AI in education,” “concerns with AI in education,” and “challenges of AI integration,” in academic databases such as PubMed, Google Scholar, ERIC, and ScienceDirect. The search initially yielded over 238 potentially relevant papers.
  • The inclusion criteria were: (1) peer-reviewed journal articles, (2) published between 2020 and 2023, (3) focused on the negative implications, drawbacks, or challenges of AI in educational settings.
  • After screening, a total of 56 papers were selected for the final analysis.
  • The content analysis process involved: (a) thorough reading, (b) inductive coding to identify recurring themes related to the potential negative impacts, (c) categorizing these into broader conceptual categories, and (d) synthesizing the findings into a theoretical model (shown in Table 1).
2. Validation of the theoretical model through survey research:
  • The derived theoretical model conceptualized the diverse challenges of AI in education and guided the design of the survey and subsequent data analysis.
  • An online survey questionnaire was developed to empirically validate and test the theoretical model, with items representing the constructs and dimensions within it.
  • The questionnaire is divided into two main sections (Appendix 1):
- Section 1: Demographic Characteristics: This section collects information about the participants' gender, current occupation, level of education, subjective AI expertise, and frequency of AI usage.
- Section 2: Concerns: This section comprises statements related to different concerns associated with AI in education as shown in Table 1. Participants are asked to rate their level of agreement with each statement using a scale ranging from 5 (strongly agree) to 1 (strongly disagree). Each concern is broken down into eight statements to capture nuances and different aspects of the issue.

### 3.2 Sampling and participation

Participants for the questionnaire were primarily selected from a university in Saudi Arabia, specifically the author's institution, to facilitate access and increase response rates. The target population comprised students, faculty members, and administrators.

By targeting individuals within the author's university community, direct access was readily available, streamlining the distribution and collection process [73]. Furthermore, focusing on a single institution helped ensure a more homogeneous sample, aligning closely with the study's specific context and objectives [74].

To engage participants, the survey questionnaire was distributed via email and personal networks within the university, aiming to reach a broader audience and encourage participation. In total, 260 individuals participated.

### 3.3 Validity and reliability of the survey questionnaire

Three experts in the field of educational research piloted the questionnaire to evaluate its relevance, accuracy, and validity. Following their input, necessary modifications were made. The revised questionnaire can be found in Appendix 1. Table 2 displays Cronbach's alpha values for each subscale and for the overall instrument. The subscales showed robust internal consistency with α ≥ 0.7. The total scale exhibited excellent reliability, with a Cronbach's alpha of 0.97, signifying a high level of internal consistency.

**Table 2:** Cronbach's alpha values for sub-scales.

Sub-scale	α
1. Loss of Human Connection	0.75
2. Data Privacy and Security Concerns	0.72
3. Algorithmic Bias and Discrimination	0.92
4. Lack of Transparency and Explainability	0.91
5. Reduced Critical Thinking and Creativity	0.90
6. Unequal Access and Technological Divide	0.90
7. Ethical Considerations in AI Use	0.84
8. Teacher Professional Development and Role	0.92
9. Dependence on AI Reliability and Maintenance	0.89
10. Implications of AI-generated Content	0.84
**Total**	0.97

## 4 Results

### 4.1 Demographic information

Table 3 presents the demographic information of the survey participants. The gender distribution shows 140 male respondents (53.8 %) and 120 female respondents (46.2 %). Regarding current occupation, the majority are students (169, 65.%), followed by faculty members (59, 22.7 %) and administrators (32, 12.3 %). In terms of educational level, most respondents hold Bachelor's degrees (190, 73.1 %), followed by Ph.D. holders (57, 21.9 %) and Master's degree holders (13, 5.%). Self-assessed AI expertise is distributed as follows: low (98, 37.7 %), medium (122, 46.9 %), and high (40, 15.4 %). Frequency of AI usage varies, with 64 respondents (24.6 %) rarely using AI, 38 (14.6 %) using it monthly, 56 (21.5 %) weekly, and 102 (39.2 %) using AI daily.

**Table 3:** Demographic information.

		N	%
Gender	Male	140	53.8
	Female	120	46.2
Current Occupation	Student	169	65.0
	Faculty	59	22.7
	Administrator	32	12.3
Education Level	Bachelor's degree	190	73.1
	Master's degree	13	5.0
	Ph.D	57	21.9
Subjective AI Expertise	Low	98	37.7
	Medium	122	46.9
	High	40	15.4
Frequency of AI Usage	Rarely	64	24.6
	Monthly	38	14.6
	Weekly	56	21.5
	Daily	102	39.2

### 4.2 Descriptive analysis

This study assessed the perceived negative impacts of AI technologies in education through a five-point Likert scale, with participants rating their agreement from 1 (strongly disagree) to 5 (strongly agree). A cutting point of 3.5, representing agreement, was employed to interpret the results.

#### 4.2.1 Loss of human connection

Table 4 addresses the potential loss of human connection in educational settings due to AI technologies. The items' mean scores, ranging from 3.53 to 3.99 for the initial five items, reflect the participants' acknowledgement of this concern. The data suggests that AI's impact on the personal ties between students and educators is cause for worry. The integration of AI technologies in education may lead to a reduced sense of support and understanding among students due to the absence of human educators. Furthermore, AI-mediated learning could compromise emotional connections and empathy within the educational experience. Additionally, an over-reliance on AI technologies may yield a learning experience that lacks personalization and individual attention.

**Table 4:** Loss of human connection.

Items	M	SD
1. AI in education may reduce personal connections	3.99	1.41
2. Students may feel less supported without human educators	3.81	1.42
3. AI learning may lack emotional connection and empathy	3.62	1.42
4. AI reliance may result in less personalized learning	3.61	1.45
5. No face-to-face interaction may impact student engagement	3.53	1.40
6. AI education may hinder interpersonal skill development	3.35	1.40
7. AI may limit collaborative learning opportunities	3.32	1.37
8. Reduced human interaction may affect student motivation and belonging	3.30	1.42

#### 4.2.2 Data privacy and security concerns

Table 5 addresses the data privacy and security issues associated with the collection and analysis of student data in AI-based education systems. A mean score of 3.59 for the first item suggests general agreement among participants regarding these concerns. This moderate mean score indicates that participants recognize the importance of data privacy within AI-driven education, implying an awareness of the potential risks and implications tied to the use of student data in an AI context.

**Table 5:** Data privacy and security concerns.

Items	M	SD
1. Collection and analysis of student data in AI-driven education raise data privacy concerns	3.59	1.45
2. Potential misuse or mishandling of student data in AI-based education is a concern	3.23	1.46
3. Data breaches in AI-driven education could have severe consequences for personal information	3.20	1.34
4. Students' privacy may be compromised due to extensive data collection in AI-driven learning	3.11	1.33
5. Stringent safeguards are needed to protect student information from unauthorized access	3.07	1.43
6. Educational institutions must ensure responsible data management when utilizing AI technologies	3.02	1.29
7. Students should have control over their own data and provide informed consent for its usage	2.93	1.24
8. Clear policies should address concerns about data security	2.92	1.23

#### 4.2.3 Algorithmic bias and discrimination

Table 6 highlights concerns about algorithmic bias and discrimination within AI-based education systems. Mean scores ranging from 3.62 to 3.91 reflect participants' views on these issues. The results suggest that systemic biases may disadvantage students from underrepresented communities within these systems, with AI algorithms potentially perpetuating discrimination against certain groups. There is a recognized need to confront potential biases in AI algorithms employed in educational decision-making, ensuring their transparency and fairness to prevent discriminatory practices.

**Table 6:** Algorithmic bias and discrimination.

Items	M	SD
1. Students from underrepresented communities may face systemic biases in AI-based education	3.91	1.38
2. AI algorithms used in education can perpetuate biases and discrimination against certain student groups	3.88	1.38
3. Potential bias in AI algorithms used for educational decision-making needs to be addressed	3.85	1.39
4. Reliance on AI systems may reinforce existing social inequalities in educational opportunities	3.84	1.40
5. AI-driven education may lead to unfair treatment or disadvantage for marginalized students	3.76	1.39
6. Lack of diversity in AI training data can result in biased outcomes for certain student demographics	3.71	1.39
7. Transparency and fairness of AI algorithms should be ensured to prevent discriminatory practices	3.69	1.43
8. Continuous monitoring and mitigation of algorithmic biases in AI-driven education is crucial	3.62	1.45

#### 4.2.4 Lack of transparency and explainability

Table 7 underscores worries related to the lack of transparency and explainability in AI-driven education, with mean scores ranging from 3.50 to 3.83. These results hint at the need for ethical guidelines to underscore the role of transparency and explainability. The ‘black-box’ nature of AI systems raises accountability and fairness concerns in educational settings. Participants stressed the importance of providing educators and students with insights into the operations of AI algorithms in education, to foster understanding and trust.

**Table 7:** Lack of transparency and explainability.

Items	M	SD
1. Ethical guidelines should emphasize transparency and explainability in AI-driven education	3.83	1.32
2. AI complexity can raise concerns about accountability and fairness in education	3.71	1.38
3. Students and educators may struggle to trust AI tools without clear explanations of functionality	3.68	1.40
4. The black-box nature of AI may limit awareness of factors influencing educational experiences	3.64	1.42
5. Educators and students should have access to information about AI algorithms used in education	3.61	1.37
6. Lack of explainability in AI hinders understanding and learning from automated decisions	3.59	1.42
7. Transparent and interpretable AI systems are necessary for confidence in educational outcomes	3.52	1.44
8. AI algorithms used in education often lack transparency, making decision-making difficult to understand	3.50	1.37

#### 4.2.5 Reduced critical thinking and creativity

Table 8 explores the potential impact of AI in education on critical thinking and creativity, with mean scores between 3.78 and 4.11 indicating participants' concerns. The findings suggest that AI systems, by providing predefined answers, may discourage students from engaging in critical analysis or creative exploration. There is a proposition that AI-based education systems should foster open-ended thinking to combat this. The reliance on AI tools could potentially reduce innovation and original thought, therefore a balance that encourages both critical thinking and creativity is essential.

**Table 8:** Reduced critical thinking and creativity.

Items	M	SD
1. Creativity could be reduced if AI provides predefined answers and restricts exploratory thinking	4.11	1.34
2. AI-mediated learning may discourage challenging assumptions and questioning information	4.07	1.31
3. AI-based education should promote open-ended and divergent thinking	3.98	1.36
4. Overreliance on AI tools may diminish students' capacity for innovative thought	3.94	1.38
5. AI-driven education may limit opportunities for independent problem-solving	3.90	1.37
6. Critical thinking skills may suffer if students primarily rely on AI for decision-making	3.83	1.39
7. Overreliance on AI may hinder the development of critical thinking skills	3.80	1.38
8. Balancing AI with activities that foster critical thinking and creativity is essential for holistic education	3.78	1.41

#### 4.2.6 Unequal access and technological divide

Table 9 addresses concerns about unequal access and the technological divide linked to AI integration in education. With mean scores from 3.68 to 4.03, the data reflect participants' views on these disparities. There's a risk that AI could widen gaps in educational opportunities due to uneven access to advanced technologies. Equitable access to AI resources is crucial to prevent the amplification of educational disparities, with educational institutions bearing responsibility for addressing this divide.

**Table 9:** Unequal access and technological divide.

Items	M	SD
1. AI integration may exacerbate existing inequalities in access to advanced technologies	4.03	1.34
2. Students without AI access may be disadvantaged in educational opportunities	3.79	1.42
3. Disadvantaged students may face barriers in accessing AI resources	3.74	1.45
4. Equal AI access should be ensured to avoid further educational inequities	3.71	1.41
5. Schools should address disparities in AI access among students	3.70	1.39
6. The technological divide between privileged students and others could widen due to AI	3.70	1.41
7. Bridging the digital divide is crucial to empower certain student populations	3.68	1.41
8. Efforts should be made to provide equitable access to AI-driven educational tools	3.35	1.40

#### 4.2.7 Ethical considerations in AI use

Table 10 delves into ethical considerations in the use of AI in education. Mean scores for the first five items, between 3.59 and 3.81, indicate a consensus on their importance. Ethical deployment of AI should be a priority, with informed consent for data use and transparent, accountable practices at the forefront of AI integration in educational contexts.

**Table 10:** Ethical considerations in AI use.

Items	M	SD
1. Educational institutions should prioritize ethical AI use and establish responsible policies	3.81	1.42
2. Algorithms should be accountable with mechanisms to address potential harm	3.79	1.42
3. Unintended consequences of AI in education should be considered and mitigated	3.61	1.45
4. Informed consent should be obtained before collecting and using student data	3.60	1.44
5. Transparency and accountability should be central when integrating AI into education	3.59	1.45
6. Ethical frameworks should guide AI development and deployment in education	3.35	1.40
7. Student, and educators should be informed about the ethical implications of AI use	3.32	1.37
8. Ownership and control of student data in AI systems need to be clearly defined	3.32	1.37

#### 4.2.8 Teacher professional development and role

Table 11 presents findings related to teachers' professional development and roles amidst AI integration in education, showing mean scores from 3.50 to 3.71. These scores reflect the significance of empowering educators for effective AI implementation in the classroom. The report supports ongoing training and development for teachers to both adapt to and skillfully integrate AI into their teaching methods. It also reaffirms the critical role teachers play in steering student learning within AI-enhanced education.

**Table 11:** Teacher professional development and role.

Items	M	SD
1. Collaboration between educators and AI can lead to impactful learning outcomes	3.71	1.38
2. Effective AI implementation in education requires adequate teacher training and support	3.71	1.39
3. Inadequate teacher training may hinder successful AI utilization in the classroom	3.69	1.43
4. Ongoing support and resources should be provided for AI-driven instructional practices	3.68	1.40
5. Successful AI integration relies on empowering teachers to embrace new approaches	3.64	1.42
6. Teachers should receive professional development for AI integration skills	3.62	1.45
7. Teacher feedback should inform the development of AI tools for education	3.59	1.42
8. Teachers' role in AI-based education involves guiding and facilitating student learning	3.50	1.37

#### 4.2.9 Dependence on AI reliability and maintenance

Table 12 touches on reliance on AI reliability and maintenance in education. Mean scores between 3.52 and 4.11 signal the weight participants place on understanding AI limitations and risks. Regular evaluation and support for AI systems are essential, and educational institutions must be equipped to handle technical issues to ensure smooth learning experiences and to avoid overdependence on AI technologies.

**Table 12:** Dependence on AI reliability and maintenance.

Items	M	SD
1. Educators and students should be aware of AI's limitations and risks in education	4.11	1.34
2. Balancing AI with alternative approaches can reduce negative consequences	3.94	1.38
3. Regular evaluations and monitoring of AI systems are necessary for reliability	3.90	1.37
4. Diversifying educational approaches beyond AI can mitigate risks	3.83	1.32
5. Backup plans should be in place for AI system failures.	3.80	1.38
6. Adequate support and maintenance should be available for AI-based education.	3.61	1.37
7. Technical issues may disrupt learning and create dependency on AI.	3.52	1.44
8. Heavy reliance on AI raises concerns about reliability	3.52	1.44

#### 4.2.10 Implications of AI-generated content

Finally, Table 13 discusses the implications of AI-generated content in education, where mean scores span from 3.52 to 4.11. Concerns are raised about the impact of AI on originality and intellectual rigor. The study underscores the necessity of ethical considerations, as well as the importance of authenticity and reliability in AI-generated materials. It also draws attention to the intellectual property aspects and the reliability of AI for tasks like automated essay grading.

**Table 13:** Implications of AI-generated content.

Items	M	SD
1. Attribution and citation are important for AI-generated content	4.11	1.34
2. AI may undermine creativity and critical thinking in content creation	3.90	1.37
3. Ethical considerations of AI-generated content need examination	3.85	1.38
4. AI-generated content may diminish the value of human input	3.83	1.32
5. Credibility and reliability of AI-generated materials may be questioned	3.80	1.38
6. AI-generated content may raise concerns about authenticity	3.64	1.42
7. Intellectual property rights of AI-generated content need addressing	3.61	1.37
8. Automated essay grading by AI raises questions about accuracy and fairness	3.52	1.44

### 4.3 Investigating the impact of demographics

A Multivariate Analysis of Variance test (MANOVA) was conducted to explore the effects of independent variables (gender, current occupation, education level, subjective AI expertise, and frequency of AI usage) on dependent variables (loss of human connection, data privacy and security concerns, algorithmic bias and discrimination, lack of transparency and explainability, reduced critical thinking and creativity, unequal access and the technological divide, ethical considerations in AI use, teacher professional development and role, dependence on AI reliability and maintenance, and implications of AI-generated content).

Results show no significant effects for gender, current occupation, education level, or subjective AI expertise. The p-values for all associated test statistics exceed 0.05, indicating no statistically significant differences in the dependent variables based on these factors.

### 4.4 Correlation matrix of the concerns regarding the implementation of AI in education

A correlational analysis using Pearson correlation coefficients was conducted to explore the relationships between the various concerns surrounding the implementation of AI in educational settings including loss of human connection, data privacy and security concerns, algorithmic bias and discrimination, lack of transparency and explainability, reduced critical thinking and creativity, unequal access and technological divide, ethical considerations in AI use, teacher professional development and role, dependence on AI reliability and maintenance, and implications of AI-generated content.

The results presented in Table 14 show that all the concerns are significantly correlated with each other at the 0.01 level (2-tailed). The strongest correlations are observed between the following pairs of concerns.

1. Dependence on AI reliability and maintenance and implications of AI-generated content (r = 0.967)
2. Lack of transparency and explainability and teacher professional development and role (r = 0.956)
3. Reduced critical thinking and creativity and dependence on AI reliability and maintenance (r = 0.900)
4. Lack of transparency and explainability and implications of AI-generated content (r = 0.902)

**Table 14:** Pearson Correlation Coefficients among concerns Influencing AI Adoption in Education (*Sig. 2-tailed*).

		C2	C3	C4	C5	C6	C7	C8	C9	C10
C1	P. Correlation	0.437**	0.502**	0.451**	0.353**	0.517**	0.844**	0.491**	0.374**	0.405**
	Sig.	0.000	0.000	0.000	0.000	0.000	0.000	0.000	0.000	0.000
	N	260	260	260	260	260	260	260	260	238
C2	P. Correlation		0.294**	0.302**	0.197**	0.296**	0.430**	0.312**	0.227**	0.243**
	Sig.		0.000	0.000	0.001	0.000	0.000	0.000	0.000	0.000
	N		260	260	260	260	260	260	260	238
C3	P. Correlation			0.798**	0.652**	0.731**	0.473**	0.886**	0.717**	0.756**
	Sig.			0.000	0.000	0.000	0.000	0.000	0.000	0.000
	N			260	260	260	260	260	260	238
C4	P. Correlation				0.743**	0.805**	0.433**	0.956**	0.882**	0.902**
	Sig.				0.000	0.000	0.000	0.000	0.000	0.000
	N				260	260	260	260	260	238
C5	P. Correlation					0.747**	0.369**	0.698**	0.900**	0.861**
	Sig.					0.000	0.000	0.000	0.000	0.000
	N					260	260	260	260	238
C6	P. Correlation						0.475**	0.810**	0.755**	0.745**
	Sig.						0.000	0.000	0.000	0.000
	N						260	260	260	238
C7	P. Correlation							0.452**	0.379**	0.403**
	Sig.							0.000	0.000	0.000
	N							260	260	238
C8	P. Correlation								0.797**	0.835**
	Sig.								0.000	0.000
	N								260	238
C9	P. Correlation									0.967**
	Sig.									0.000
	N									238

C1: Loss of Human Connection. C2: Data Privacy and Security Concerns. C3: Algorithmic Bias and Discrimination. C4: Lack of Transparency and Explainability. C5: Reduced Critical Thinking and Creativity. C6: Unequal Access and Technological Divide. C7: Ethical Considerations in AI Use. C8: Teacher Professional Development and Role. C9: Dependence on AI Reliability and Maintenance. C10: Implications of AI-generated Content.

The weakest correlations, although still significant, are found between.

1. Reduced critical thinking and creativity and data privacy and security concerns (r = 0.197)
2. Data privacy and security concerns and dependence on AI reliability and maintenance (r = 0.227)
3. Data privacy and security concerns and implications of AI-generated content (r = 0.243)

These findings imply that concerns about AI adoption in education are interconnected, with certain concerns more strongly associated than others. Addressing one area may positively impact others, underscoring the importance of a comprehensive strategy in AI integration within educational settings.

## 5 Discussion

The adoption of AI technologies in education is a transformative movement that brings rewarding possibilities alongside formidable challenges. As illustrated by recent studies [8], the deployment of AI in educational settings boasts prospects for personalized learning pathways and efficient administrative operations. However, this technological infusion also introduces complex issues that warrant careful examination and strategic intervention.

A prominent concern highlighted in scholarly debate involves the potential erosion of the human touch in learning environments [[15], [16], [17], [18], [19], [20], [21]]. The reduction in direct interpersonal interactions could diminish the relational quality that is fundamental to the educational journey. Therefore, it is essential to implement AI as an extension of human educators rather than a substitute, ensuring that technology adds value to the student-teacher dynamic rather than detracting from it. This approach emphasizes the creation of AI solutions that are not only intellectually engaging but emotionally intelligent, fostering an educational atmosphere where personalized attention flourishes.

In parallel, the concerns surrounding data privacy and security take precedence as AI systems often operate on vast repositories of personal student information [[22], [23], [24], [25]]. The ethical implications of data handling in AI systems necessitate robust frameworks for governance, emphasizing transparency and control for individuals over their own data. Moreover, as AI models are gradually integrated into educational processes, it is paramount to enforce stringent standards that protect against unauthorized data use and breaches, thereby upholding student privacy and trust.

Issues of algorithmic bias and discrimination present another significant challenge [[26], [27], [28], [29], [30], [31], [32], [33]]. If not vigilantly addressed, these biases can insidiously propagate existing disparities within educational systems. Therefore, it is critical to adopt a transparent algorithmic framework that is regularly audited for biases and corrected accordingly, ensuring fair and equal treatment for all students.

Transparency and explainability in AI systems are further marred by technical complexities [[34], [35], [36], [37], [38], [39], [40]]. Complicated algorithms can be perceived as opaque and unaccountable, which could lead to resistance from those it aims to serve. Demystifying these systems via explainable AI should therefore be a priority, so that not only are decision-making processes clear, but learners and educators alike can understand and trust the AI tools with which they engage.

Moreover, there is an observed potential for AI to impact learners' critical thinking and creative capacities [[41], [42], [43]]. When AI systems are designed to offer straightforward solutions or paths of least resistance, they may inadvertently dampen the incentives for in-depth problem-solving and outside-the-box thinking. It is vital for educational AI applications to encourage exploration and inquiry, enhancing learners' analytical abilities and fostering innovative thought processes.

The digital divide reflects yet another trench of inequality, as unequal access to AI technologies can exacerbate differences in educational quality and outcomes [[44], [45], [46], [47], [48]]. As AI becomes more embedded in educational curricula, it is imperative to advance initiatives that promote equitable accessibility, ensuring that all students, regardless of their socioeconomic status, can benefit from AI-enabled educational tools.

Ethical considerations such as informed consent, data ownership, and algorithmic accountability play a pivotal role in responsible AI integration [[49], [50], [51], [52], [53], [54], [55]]. These require the establishment of a well-defined ethical framework that places human rights at the forefront of AI in education, ensuring that these sophisticated systems serve as a beneficent force rather than a source of exploitation.

Supporting educators through tailored professional development programs is essential to marshal the efficacious use of AI in education [[56], [57], [58], [59], [60]]. Teachers must be provided with the knowledge and tools necessary to supplement their expertise with AI resources, creating a symbiotic relationship between human judgment and algorithmic efficiency.

Concerns about the reliability and consistent maintenance of AI systems highlight the importance of infrastructural integrity in AI's educational utility [[61], [62], [63], [64], [65], [66]]. Ensuring the dependability of these systems is non-negotiable, given that technological failure could lead to substantial disruptions in the learning process.

The implications of AI-generated content within the academic sphere also stimulate discourse on authenticity and intellectual property [41,[67], [68], [69], [70], [71]]. Balancing the innovative contributions of AI with the revered traditions of academic integrity and human creativity is an ongoing dialogue that underscores the need for clear guidelines and ethical practices.

Further, the findings from the correlation analyses offer insights into the multifaceted issues that educational institutions face when integrating AI technology into their systems. The interconnectedness of the concerns, as evidenced by the significant correlations, suggests that these factors do not exist in isolation. Instead, they influence and amplify each other in complex ways.

For example, the strong correlation between the dependence on AI reliability and the implications of AI-generated content indicates that ensuring the reliability and maintenance of AI systems could also mitigate concerns regarding the quality and credibility of AI-generated materials. Similarly, improving transparency and explainability can have a two-fold effect by not only making AI systems more comprehensible and accountable but also by aiding teacher professional development, thereby enabling educators to better facilitate AI integration in their teaching.

The weaker, yet significant, correlations, such as between reduced critical thinking and data privacy and security concerns, imply that there are broad impact zones where improvements in one area could potentially have ripple effects on the other, albeit to a lesser degree. In this case, enhancing data security measures might indirectly bolster critical thinking by creating a safer environment for open-ended discussions and exploration without the worry of data misuse.

These interrelationships affirm that singular, piecemeal solutions are unlikely to be effective [2]. Rather, holistic strategies in AI policy-making are required, considering the domino effects within the AI in education ecosystem, for a robust, equitable, and effective environment. By doing so, they can create a more robust, equitable, and effective educational environment that capably leverages the benefits of AI while minimizing its risks and unintended consequences.

## 6 Conclusion and implications

The integration of AI technology in education can indeed personalize learning experiences, but it also raises concerns about human connection, data privacy and security, algorithmic bias, transparency, critical thinking, access equity, ethical issues, teacher development, reliability, and the consequences of AI-generated content.

This study sheds light on the complex interplay of factors influencing AI integration in education. Understanding these relationships enables educators, policymakers, and stakeholders to devise targeted strategies to navigate the challenges and capitalize on the opportunities presented by AI technologies in educational contexts.

While AI holds immense potential in education, its integration must be carefully navigated to uphold core values and mitigate risks. A balanced approach should strive to preserve the human connections that are vital for emotional development, while robustly safeguarding data privacy and security. Proactive measures are needed to combat algorithmic biases, increase transparency around decision-making processes, and promote environments that nurture critical thinking alongside technological proficiency. Equitable access must be a priority to bridge digital divides. Ethical considerations surrounding student rights, well-being, content authenticity, intellectual property, and fair assessment cannot be overlooked. Continuous investments in teacher training, contingency planning, consistent support, and rigorous evaluations are imperative. By thoughtfully addressing these multifaceted implications, the responsible adoption of AI can enhance educational experiences while upholding the integrity of the learning ecosystem.

## 7 Limitations and future directions

This study is subject to several limitations. First, the sample size of 260 participants may not fully capture the diversity necessary for broader generalizability. Thus, a larger and more diverse sample could offer a wider perspective on the concerns regarding AI integration in education. Furthermore, the findings may not be universally applicable across various educational settings or populations due to the role of contextual factors in shaping perceptions and concerns.

These limitations underscore the need for further research to bridge these gaps and deepen our understanding of AI's implications in education. Future studies should examine the long-term effects of AI on teaching and learning outcomes, as well as devise innovative strategies to promote ethical AI usage and ensure equitable access in educational environments.

## Funding statement

This research did not receive any specific grant from funding agencies in the public, commercial, or not-for-profit sectors.

## Data availability

The data associated with this study has not been uploaded into a publicly available repository. However, the data can be provided upon a reasonable request.

## Ethics declarations

Ethics approval was obtained from the Faculty of Education Research Committee prior to conducting the research, and written informed consent was obtained from the participants.

## CRediT authorship contribution statement

**Abdulrahman M. Al-Zahrani:** Writing – review & editing, Writing – original draft, Visualization, Validation, Supervision, Software, Resources, Project administration, Methodology, Investigation, Funding acquisition, Formal analysis, Data curation, Conceptualization.

## Declaration of competing interest

The authors declare that they have no known competing financial interests or personal relationships that could have appeared to influence the work reported in this paper.

## Figures and Tables

**Table:** 

Gender	1. Male 2. Female
Current Occupation	1. Student 2. Faculty 3. Administrator
Education	1. Bachelor's degree 2. Master's degree 3. Ph.D.
Subjective AI Expertise	1. Low perceived expertise 2. Medium perceived expertise 3. High perceived expertise
Frequency of AI Usage	1. Rarely 2. Monthly 3. Weekly 4. Daily

**Table:** 

A. Loss of Human Connection
1.1. AI technologies in education may diminish the personal connection between students and educators.
1.2. The absence of face-to-face interaction in the learning process could negatively impact students' engagement.
1.3. AI-mediated learning might lead to a lack of emotional connection and empathy between students and educators.
1.4. Students' motivation and sense of belonging could be affected due to reduced human interaction in education.
1.5. AI-based education might hinder the development of interpersonal skills among students.
1.6. The reliance on AI technologies may result in a less personalized and individualized learning experience.
1.7. AI in education may limit opportunities for collaborative learning and group discussions.
1.8. Students might feel less supported and understood without the presence of human educators.
B. **Data Privacy and Security Concerns**
2.1. The collection and analysis of student data in AI-driven education raise concerns about data privacy.
2.2. There is a need for stringent safeguards to protect student information from unauthorized access.
2.3. The potential misuse or mishandling of student data in AI-based education is a significant concern.
2.4. Students' privacy may be compromised due to the extensive data collection involved in AI-driven learning.
2.5. Clear policies should be in place to address concerns about data security in AI-powered education.
2.6. Students should have control over their own data and be able to provide informed consent for its usage.
2.7. Educational institutions must ensure responsible data management practices when utilizing AI technologies.
2.8. Data breaches in AI-driven education could have severe consequences for students' personal information.
C. **Algorithmic Bias and Discrimination**
3.1. AI algorithms used in education can perpetuate biases and discrimination against certain student groups.
3.2. The reliance on AI systems may reinforce existing social inequalities in educational opportunities.
3.3. There is a need to address the potential bias in AI algorithms used for educational decision-making.
3.4. AI-driven education may lead to unfair treatment or disadvantage for marginalized student populations.
3.5. Students from underrepresented communities may face systemic biases in AI-based educational systems.
3.6. The lack of diversity in AI training data can result in biased outcomes for certain student demographics.
3.7. It is crucial to continuously monitor and mitigate algorithmic biases in AI-driven education.
3.8. The transparency and fairness of AI algorithms should be ensured to prevent discriminatory practices.
D. **Lack of Transparency and Explainability**
4.1. AI algorithms used in education often lack transparency, making it difficult to understand how decisions are made.
4.2. The opacity of AI systems can raise concerns about accountability and fairness in educational processes.
4.3. Students and educators may struggle to trust AI-based educational tools without clear explanations of their functionality.
4.4. The lack of explainability in AI systems hinders students' ability to understand and learn from automated decisions.
4.5. The black-box nature of AI algorithms may limit students' awareness of the factors influencing their educational experiences.
4.6. Transparent and interpretable AI systems are necessary for students to have confidence in their educational outcomes.
4.7. Educators and students should have access to information about the workings of AI algorithms used in education.
4.8. Ethical guidelines should emphasize the importance of transparency and explainability in AI-driven education.
E. Reduced **Critical Thinking and Creativity**
5.1. Overreliance on AI technologies may hinder students' development of critical thinking skills.
5.2. AI-driven education might limit opportunities for students to engage in independent problem-solving.
5.3. Creativity may be stifled if AI systems provide predefined answers and restrict exploratory thinking.
5.4. Students may become overly reliant on AI tools, diminishing their capacity for innovative and original thought.
5.5. AI-mediated learning may discourage students from challenging assumptions and questioning information.
5.6. Critical thinking skills may suffer if students primarily rely on AI for decision-making and problem solving.
5.7. AI-based education should promote opportunities for students to engage in open-ended and divergent thinking.
5.8. Balancing AI technologies with activities that foster critical thinking and creativity is essential for holistic education.
F. **Unequal Access and Technological Divide**
6.1. The integration of AI in education may exacerbate existing inequalities in access to advanced technologies.
6.2. Students from disadvantaged backgrounds might face barriers in accessing AI resources and infrastructure.
6.3. The technological divide between privileged and disadvantaged students could widen due to AI implementation.
6.4. Equal access to AI tools and resources should be ensured to avoid further educational inequities.
6.5. Students without access to AI technologies may be at a disadvantage in terms of educational opportunities.
6.6. Bridging the digital divide is crucial to prevent the marginalization of certain student populations.
6.7. Schools and educational institutions should address the disparities in AI access among students.
6.8. Efforts should be made to provide equitable access to AI-driven educational tools and resources.
G. **Ethical Considerations in AI Use**
7.1. Informed consent should be obtained before collecting and using student data in AI-based education.
7.2. The ownership and control of student data in AI systems need to be clearly defined.
7.3. Algorithms used in education should be accountable, and there should be mechanisms to address potential harm.
7.4. Ethical frameworks should guide the development and deployment of AI technologies in educational settings.
7.5. Potential unintended consequences of AI in education should be carefully considered and mitigated.
7.6. Students, parents, and educators should be informed about the ethical implications of AI use in education.
7.7. Educational institutions should prioritize the ethical use of AI and establish policies to ensure responsible practices.
7.8. Transparency and accountability should be central principles when integrating AI into educational contexts.
H. **Teacher Professional Development and Role**
8.1. Effective implementation of AI technologies in education requires adequate teacher training and support.
8.2. Teachers should receive professional development opportunities to enhance their AI integration skills.
8.3. Inadequate teacher training may hinder the successful utilization of AI tools in the classroom.
8.4. Teachers' role in AI-based education should involve guiding and facilitating student learning experiences.
8.5. Collaborative efforts between educators and AI technologies can lead to more impactful learning outcomes.
8.6. Ongoing support and resources should be provided to educators to adapt to AI-driven instructional practices.
8.7. Teacher feedback and insights should inform the development and improvement of AI tools for education.
8.8. The successful integration of AI in education relies on empowering and enabling teachers to embrace new pedagogical approaches.
I. **Dependence on AI Reliability and Maintenance**
9.1. Heavy reliance on AI systems for educational tasks raises concerns about their reliability.
9.2. Technical issues and system failures may disrupt learning processes and create dependency on AI technologies.
9.3. Adequate support and maintenance should be available to address potential disruptions in AI-based education.
9.4. Diversifying educational approaches beyond AI can mitigate the risks associated with reliance on a single technology.
9.5. Backup plans should be in place to ensure continuity in learning in case of AI system failures.
9.6. Regular evaluations and monitoring of AI systems' performance are necessary to maintain their reliability.
9.7. Educators and students should be aware of the limitations and potential risks of AI technologies in education.
9.8. Balancing the benefits of AI with alternative approaches can reduce the negative consequences of reliance on AI systems.
J. Implications of AI-generated Content
10.1. AI-generated educational content may raise concerns about the authenticity of the materials.
10.2. The use of AI for automated essay grading raises questions about the accuracy and fairness of assessments.
10.3. The intellectual property rights of AI-generated educational content need to be carefully addressed.
10.4. The value of human input in educational processes may be diminished when AI generates content.
10.5. Educators and students may question the credibility and reliability of AI-generated educational materials.
10.6. The role of creativity and critical thinking in educational content creation may be undermined by AI.
10.7. Proper attribution and citation practices become important when utilizing AI-generated content.
10.8. The ethical considerations of using AI-generated content in education require careful examination.

## Appendix 1

Survey Questionnaire.

Unveiling the Shadows: Beyond the Hype of AI in Education.

### 2. Concerns

Please rate the following statements based on your level of agreement:

Strongly Disagree (1), Disagree (2), Neutral (3), Agree (4), Strongly Agree (5).
